# Methane emissions from rice paddies are regulated by carbon availability and soil pH along a mean annual temperature gradient

**DOI:** 10.1038/s41598-026-43940-8

**Published:** 2026-03-19

**Authors:** Dai Yusong, Cao Jiawei, Li Huabin, Hu Jinli, Liu Guangcheng, Su Ronglin, Wu Xian, Wang Yan, Hu Ronggui

**Affiliations:** 1 College of Resources and Environment, Huazhong Agricultural University, No. 1 Shizishan Street, Hongshan District, Wuhan, 430070 Hubei The People’s Republic of China; 2 Key Laboratory of Green Utilization of Critical Non-metallic Mineral Resources, Ministry of Education, Wuhan University of Technology, Wuhan, 430070 The People’s Republic of China; 3 School of Resources and Environmental Engineering, Wuhan University of Technology, Wuhan, 430070 The People’s Republic of China; 4 School of Life Sciences, Fudan University, Shanghai, 200438 The People’s Republic of China; 5 State Key Laboratory of Soil and Sustainable Agriculture, Institute of Soil Science, Chinese Academy of Sciences, Nanjing, 210008 The People’s Republic of China

**Keywords:** Paddy soil, Rice field, Methane emission, Climate–soil interactions, Soil organic carbon fractions, Biogeochemistry, Climate sciences, Ecology, Ecology, Environmental sciences, Microbiology

## Abstract

Rice paddies are a major anthropogenic source of atmospheric methane (CH₄), yet the spatial pattern and underlying mechanism of CH₄ emissions from rice paddies across climatic gradients remain poorly understood. We collected and incubated 30 flooded paddy soils spanning tropical to temperate regions of China, quantified CH₄ emissions and explored their soil and microbial drivers. We discovered that cumulative CH₄ emissions exhibited pronounced geographical variability, with higher emissions in tropical soils (0.18–10.75 mg kg^−1^) than in temperate soils (0.07–0.17 mg kg^−1^), and were primarily regulated by dissolved organic carbon (DOC), DOC accounted for 36.0% of the variance in cumulative CH₄ emissions. Peak CH₄ emission rates were jointly influenced by DOC and microbial biomass carbon, together they explained 24.4% of the variance in peak CH₄ emission rates. The timing of peak emissions was governed by the slow degradation of particulate organic carbon (POC), POC accounted for 13.8% of the variance in the timing of peak emissions. Structural equation modeling (SEM) further revealed that soil pH and mean annual temperature (MAT) could indirectly regulate cumulative CH₄ emissions through affecting the accumulation of labile carbon and nitrogen pool, the model explained 63% of the spatial variation in cumulative CH₄ emissions in total. The indirect effect of MAT was 0.20, and the indirect effect of soil pH was − 0.26. These results highlight the critical role of climate–soil-microbe interactions in shaping regional patterns of methane emissions from rice paddies and provide mechanistic insights for improving CH₄ emission predictions under future climate change.

**Supplementary Information:**

The online version contains supplementary material available at 10.1038/s41598-026-43940-8.

## Introduction

As the second most important anthropogenic greenhouse gas after carbon dioxide, methane (CH₄) displayed a considerable global warming potential approximately 32 times greater than CO₂ over a span of 100 year, significantly contributing to near-future climate forcing^1,2^. Since the preindustrial era, rising atmospheric CH_4_ concentrations have contributed approximately 0.6 °C to global mean surface warming, with the updated radiative forcing estimated at ~ 0.61 W m^−2^^3^ (Navik et al. 2021). Among terrestrial ecosystems, flooded rice paddies represent one of the largest anthropogenic sources of CH₄ due to sustained anaerobic soil conditions that favor methanogenesis^4,5^. Globally, paddy soils occupy extensive lowland agricultural regions and contribute an estimated 30 Tg CH₄ yr^−1^ during the 2010–2019 period, corresponding to approximately 8% of total anthropogenic methane emissions^6^. China hosts one of the largest rice cultivation areas worldwide, exceeding 30 million hectares in recent years, underscoring its disproportionate importance in regional and global CH₄ budgets, the annual methane emissions from Chinese paddy fields are 8.3 Tg, accounting for 33.1% of the total global methane emissions from paddy fields^7,8^.

The magnitude of CH_4_ emissions is strongly regulated by irrigation practices, organic matter inputs, and soil biogeochemical properties, leading to substantial spatial heterogeneity from tropic to temperate zones^9–12^. Given the ongoing trend for global warming and projected increases in future rice demand, it is critical to improve our understanding of the methane emissions and mechanism in Chinese paddy soils across climatic conditions to further refine global CH₄ inventories and develop effective mitigation strategies^13^.

The production of CH₄ in paddy soils generally originates from the anaerobic decomposition of soil organic carbon (SOC) by methanogenic archaea under flooded environment^4^. As the principal substrate for methanogenesis, SOC directly regulates CH₄ emission rates and thereby further influencing greenhouse gas feedback^14^. SOC represents a complex assemblage of compounds with contrasting chemical composition and turnover times, containing both stable recalcitrant carbon pools and fast cycling labile carbon pools such as dissolved organic carbon (DOC), particulate organic carbon (POC), and microbial biomass carbon (MBC) being more readily available for microbial metabolism and thus play vital roles in regulating biogeochemical processes^15^. Recent evidence has shown that these soil labile carbon fractions were closely linked to GHG emissions in paddy fields by serving readily available substrates for methanogenic activity and shaping the balance between methane production and oxidation^16^. Therefore, spatial variation in the distribution and composition of these labile SOC pools can contribute to substantial differences in CH_4_ flux, particularly among flooded systems under contrasting soil properties, management practices, and climatic conditions^17^. Consequently, exploring the relationships between labile SOC fractions and CH_4_ emissions is essential for understanding the spatial heterogeneity and mechanistic controls of methane emissions in paddy fields.

Climate factors, especially temperature and precipitation regimes, further exert substantial influence on SOC dynamics and methane emissions through regulating microbial activities^18–20^. Elevated temperature generally accelerates organic matter decomposition and enhances substrate availability, therefore, spatial variation in MAT could shape broad geographical patterns in carbon turnover and microbial activity, further underpin large-scale geographical paddy soil CH_4_ emission pattern. Recent experimental evidence further indicates that warming alters the balance of methanogenic and methanotrophic communities in rice paddies, often resulting in enhanced CH₄ emissions under warmer conditions^21^. However, despite recognition of these climate controls, existing studies remain largely region-specific or focus on single environmental drivers, and there is still limited mechanistic understanding of how climatic gradients interact with soil physicochemical properties and labile SOC fractions to regulate methane emissions at regional scales.

Indeed, the dominant SOC fractions controlling CH₄ emissions remain inconsistent among studies, likely due to interactions among soil carbon availability, microbial activities, and environmental properties such as soil pH, moisture regime, and climatic gradients^17,22,23^. For instance, some studies highlighted DOC as the principal determinant of CH₄ emissions, it promotes methane emissions by enhancing the activity and growth of methanogens and accelerating the production and accumulation of methane precursors.^24^. Nevertheless, existing research identified significant role of MBC or soil properties in modulating methane production mechanism, higher MBC content usually indicates stronger microbial activity and more active methanogenesis, which promotes the decomposition of organic substrates and the production of methane^25,26^. POC regulates methane emissions indirectly by continuously supplying fermentable substrates through slow microbial degradation,soils with higher POC content generally exhibit delayed peak methane emissions due to the slow decomposition characteristics of POC^27,28^. Recent work further suggests that labile organic carbon fractions and associated microbial processes together regulate greenhouse gas emissions pathways from paddy soils, with variability in C substrate availability and microbial responses contributing to divergent results from flooding to drying conditions^11,12^. Moreover, the influence of methanogenic community structure and activity can be mediated by SOC and soil properties, indicating coupled biotic–abiotic interactions may underpin spatial inconsistencies in labile carbon controls on CH₄ fluxes^29^. Global syntheses also reveal that the relative importance of SOC and associated variables varies with water regime, organic amendments, and soil conditions, further explaining why different studies report contrasting dominant controls on methane emissions^30^. These discrepancies suggest the spatial variation and context-dependency of labile carbon pools regulated by climate and soil properties. Consequently, elucidating the relative importance of these labile carbon pools across spatial climatic gradients remains an unresolved challenge.

Despite this, existing studies have largely been region-specific or focused on individual environmental factors^28^, there remains limited mechanistic understanding of how climatic gradients interact with soil physicochemical properties and labile carbon fractions to govern CH₄ emissions at regional scales^17,25^. To address these uncertainties, we collected 30 representative paddy soil samples spanning from tropical to temperate zones across China and conducted incubation experiments to systematically quantify methane emissions and underlying mechanisms. By evaluating the interactions among climate, soil physicochemical properties, microbial activities, and how they regulate CH₄ fluxes, this study aims to elucidate the spatial pattern of paddy soil methane emissions across tropical and temperate zones and to clarify how climate–soil interactions regulate this spatial variability.

## Data and methods

### Characteristics of the sampling soils

**Fig. 1:**
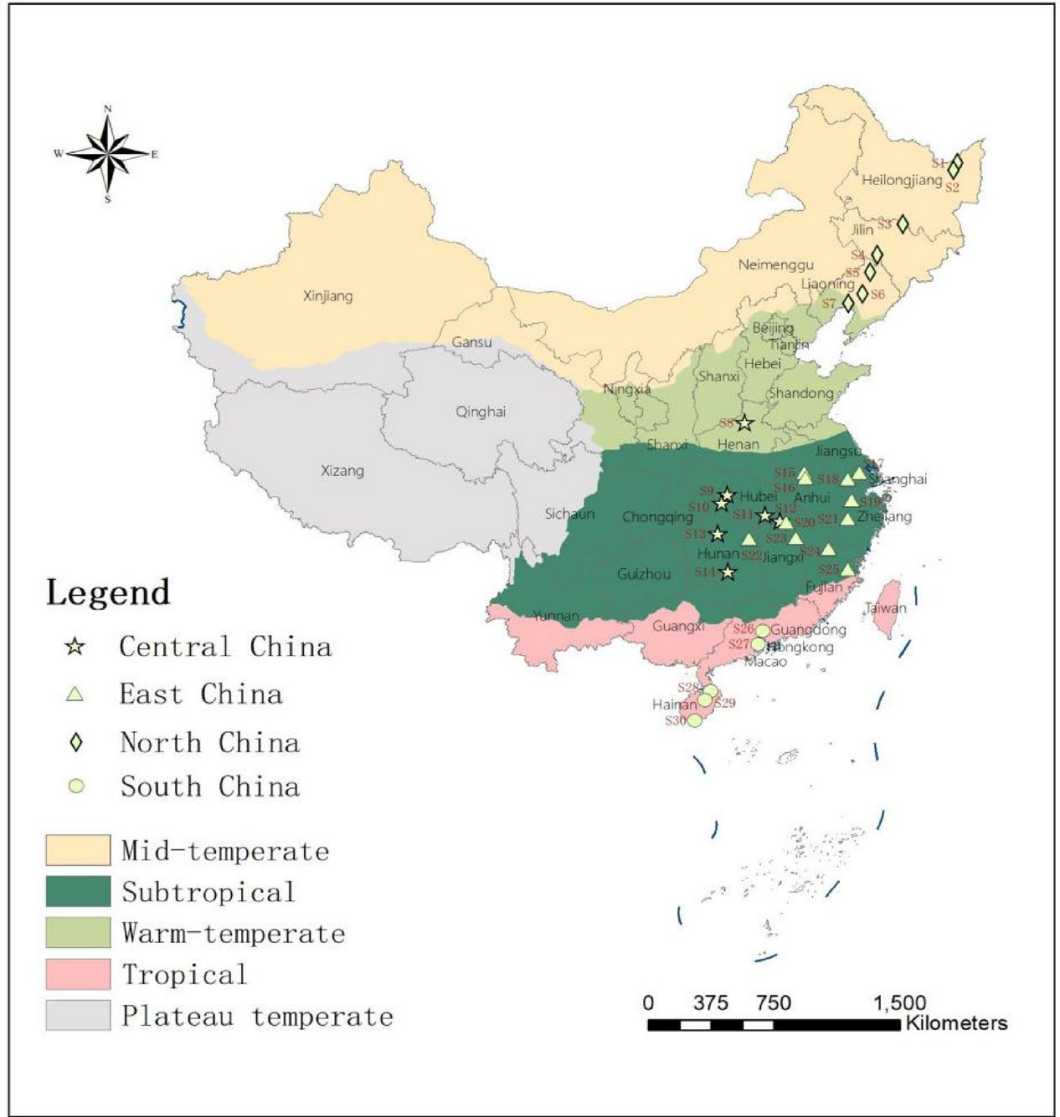
Sampling soil distribution across China’s major rice cultivation regions.

Thirty representative soil samples were taken from paddy fields along a south-north transect (18.32° N–47.53° N) across China’s major rice cultivation regions during June to December 2021 (Fig. 1 and Table 1). All samples were obtained from 0 to 20 cm soil layer, used for the analysis on total carbon (TC), total nitrogen (TN), SOC, pH, mechanical composition, as well as mineral-associated carbon (MAOC) and POC. Before the experiment, the sampled soils were air-dried naturally. We then removed plant residues and stones, ground the soils to pass through a 2-mm sieve and stored them at room temperature. More detailed information on soil sampling is summarized in Table 1. Abbreviations and their full names are detailed in the Supporting information Table 1.

**Table 1:** Characteristics of the sampling paddy soils.

Climate and Region		Sample sites		Latitude	Longitude	Average annual temperature (℃)	Average annual rainfall (mm)
Climate zone	Area	Full name	No				
Mid-temperate	North China	Jiamusi1	S1	47.53°N	132.48°E	2.9	629.62
		Jiamusi2	S2	47.18°N	131.99°E	3.4	594.89
		Haerbin	S3	44.85°N	127.14°E	3.5	820.4
		Changchun	S4	43.45°N	124.73°E	6.9	762.81
		Tielin	S5	42.54°N	123.97°E	7	1062.18
		Liaoyang	S6	41.44°N	123.10°E	7.9	882.08
		Panjin	S7	41.05°N	121.96°E	8.3	737.97
Warm-temperate	Central China	Xinxiang	S8	35.07°N	113.75°E	14.00	485.47
Subtropical	Central China	Jinmen	S9	31.11°N	112.21°E	17.45	823.85
		Yichang	S10	30.64°N	111.82°E	16.6	806.16
		Xianning	S11	29.79°N	114.48°E	16.8	1116.38
		Huanggang	S12	29.39°N	115.32°E	17.6	979.82
		Changde	S13	28.93°N	111.46°E	17.49	1198.62
		Yongzhou	S14	26.76°N	111.88°E	18.2	1299.2
	East China	Hefei2	S15	31.96°N	117.21°E	15	870.67
		Hefei1	S16	31.66°N	117.25°E	15.4	843.76
		Suzhou	S17	31.55°N	120.70°E	15.7	942.07
		Wuxi	S18	31.29°N	119.91°E	16.2	1023.58
		Hangzhou	S19	30.08°N	119.93°E	16.3	1356.75
		Jiujiang	S20	29.28°N	115.72°E	16.8	1198.88
		Jinhua	S21	29.07°N	119.51°E	17.9	1569.22
		Yichun	S22	28.54°N	113.33°E	17.5	1341.26
		Nanchang	S23	28.34°N	116.19°E	17.7	1709.98
		Nanping	S24	27.50°N	118.07°E	18.3	1882.43
		Fuzhou	S25	26.23°N	119.07°E	19.5	1841.15
	South China	Guangzhou	S26	23.25°N	113.64°E	22.2	2234.95
		Zhongshan	S27	22.53°N	113.33°E	23.45	1698.48
Tropical	South China	Haikou	S28	19.99°N	110.44°E	23.8	1623.11
		Tunchang	S29	19.46°N	110.09°E	23.5	1536.04
		Sanya	S30	18.32°N	109.48°E	25.4	1438.18

### Incubation experiment

Indoor incubation experiments were conducted using 30 paddy soil samples collected across tropical to temperate climatic zones in China to investigate CH_4_ emission characteristics and their driving factors. Experiments included soil activation, establishment of culture conditions, gas sampling and measurement of SOC. Fresh soil equivalent to 30 g dry weight was weighed into 150 mL serum bottles, adjusted to 40% gravimetric water content, and pre-incubated at 25 °C in the dark for 7 days to allow soil microbial acclimation to the artificial cultivation environment. Soil microorganisms and enzymes are most active at 18–25 °C, with near-peak activity at 25 °C. This temperature range ensures stable microbial metabolism, residue decomposition, and methane production, while avoiding inhibition by low temperatures (< 15 °C) or excessive substrate consumption at high temperatures (> 30 °C), allowing reliable assessment of key soil processes^4^. After the activation period, the headspace was flushed with synthetic air (N_2_:O_2_ = 4:1) and flooding conditions were established at a soil-to-water ratio of 1:1.5 for incubation at 25 °C. After headspace gas replacement with pure gas, the serum bottles were sealed and transferred to a constant-temperature incubator. Each soil sample was prepared in triplicate. Gas samples were collected at 1, 3, 5, 7, 10, 14, 21, 28, 35, and 42 days of incubation. At each sampling time, the headspace was replenished with fresh synthetic gas and resealed until the next collection. Gas samples were obtained using a syringe equipped with a fine needle that penetrated the rubber septum, with 10 mL of headspace gas collected after thorough mixing by repeated piston movements. CH_4_ concentrations were determined by gas chromatography (Agilent 7890A) equipped with a flame ionization detector (FID). A portion of the activated fresh soil was reserved for subsequent determination of DOC, POC, and MBC.

### Determination of soil physical and chemical properties

Soil pH was measured in a 1:2.5 (w:v) soil–water suspension with a pH meter. Soil texture was determined by the hydrometer method following sedimentation of a dispersed soil–water suspension. SOC and TN were determined using an elemental analyzer (Vario EL III; Elementar, Langenselbold, Germany). Soil available N (NH_4_-N and NO_3_-N) was extracted from moist soil using 2 M KCl solution (with a soil–solution ratio of 1:5) and was analyzed on a continuous-flow autoanalyzer. MBC was determined by chloroform fumigation, measured with a total organic carbon (TOC) analyzer. Soil MAOC and POC were determined using the sodium hexametaphosphate ((NaPO₃)₆) dispersion-sieving method and analyzed using a TOC analyzer (Cambardella and Elliott 1993).

### Calculation on the cumulative CH_4_ emissions

The cumulative CH_4_ emissions during each sampling interval were calculated from the headspace CH_4_ concentration using following Eq. 1:

1 $${\mathrm{E}} = { rho } \times \left( {{273}/{\mathrm{T}}} \right) \times \left( {{\mathrm{V}}/{\mathrm{m}}} \right) \times {\mathrm{c}} \times { alpha }$$

where *E* represents the cumulative CH_4_ emission (unit: mg C·kg^−1^), and *ρ* is the CH_4_ density at standard conditions (273 K, 1.013 × 10^5^ Pa = 0.717 kg·m^−3^); T denotes the incubation temperature (K), *V* and *m* stand for the headspace volume (L) and the soil mass (g), respectively; *c* and *α* are the measured CH_4_ concentration (mg·kg^−1^) and the conversion factor from CH_4_ to C (12/16), respectively.

The CH_4_ emission rate for each interval was determined using Eq. 2:

2 $${\mathrm{R}} = { rho } \times \left( {{273}/{\mathrm{T}}} \right) \times \left( {{\mathrm{V}}/{\mathrm{m}}} \right) \times {\mathrm{c}} \times { alpha }/{\mathrm{t}}$$

where *R* signifies the CH_4_ emission rate (unit: mg C·kg^−1^·d^−1^), and t refers to the incubation duration (days). The peak CH_4_ emission rate(PCE) was operationally defined as the maximum observed rate across all sampling intervals, with the time to peak recorded as the sampling day when this maximum occurred.

### Data analysis

To identify the factors influencing CH_4_ emissions and to assess the strength and direction of relationships between emission characteristics and environmental variables, we conducted Pearson correlation analyses using software SPSS 26 between three emission parameters (cumulative CH_4_ emissions, peak emission rate, and time to peak) and the relative parameters, such as MAT, POC, DOC, MBC, mineral-associated organic carbon (MAOC), soil pH, soil TC, TN, soil texture fractions (silt, clay and sand contents), inorganic nitrogen forms (NH_4_^+^ and NO_3_^−^), and soil C/N ratio. These analyses provided an initial assessment of potential factors driving the dynamics of methane emissions.

To further explore the mechanism of CH_4_ emissions, Pearson correlation and partial least squares (PLS) regression were employed to evaluate the relative importance of each variable in predicting emission parameters. PLS regression is particularly appropriate for analyzing datasets with multicollinearity among predictors. Variables were standardized before PLS modeling, and the importance of each predictor was evaluated using Variable Importance in Projection (VIP) scores. Variables possessing VIP (Variable Importance in Projection) scores greater than 1 considered significant explanatory factors and their relationships with emission parameters were further visualized using linear models. To meet the assumptions of parametric statistical tests, all predictors except pH were log10-transformed to approximate normal distributions. Pearson correlation analyses and linear modeling were performed using Origin software. To explore a more comprehensive evaluation of direct and indirect effects on cumulative CH_4_ emissions, we implemented structural equation modeling (SEM) using Smart-PLS 4. This multivariate approach allowed simultaneous examination of multiple causal pathways, with model fit assessed through R^2^ values.

## Results

### Spatial variation in soil properties and SOC fractions

**Fig. 2:**
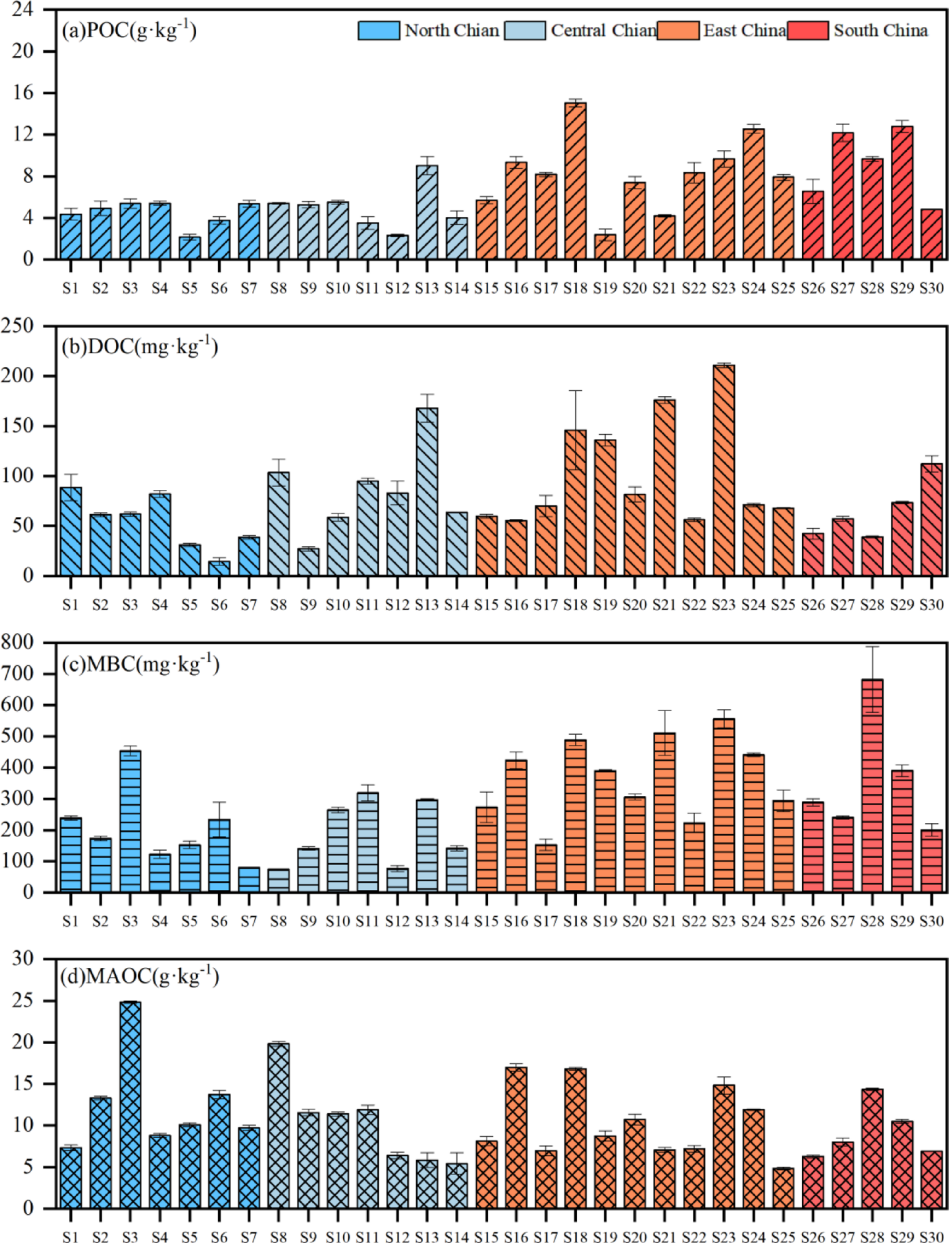
The content of SOC fractions of paddy soils.

Overall, paddy soils exhibited distinct spatial heterogeneity in physicochemical properties and SOC fractions across northern to southern China (Table. 2; Fig. 2). Generally, physicochemical properties such as TC, TN, C/N ratios, soil pH and NH₄⁺ and NO_3_^−^ contents varied substantially from northern to southern China, ranging from 8.85 to 31.86 g·kg^−1^, 0.88 to 3.24 g·kg^−1^, 8.14 to 14.02, 7.78 to 5.43, 0.91 to 24.49 mg·kg^−1^ and 0.21 to 28.11 mg·kg^−1^, respectively. On average, TC, NH₄⁺ and NO_3_^−^ contents showed a moderate increasing trend from northern (16.96 g·kg^−1^; 0.91 mg·kg^−1^; 0.21 mg·kg^−1^) to southern (18.42 g·kg^−1^; 24.49 mg·kg^−1^; 28.11 mg·kg^−1^) China. In contrast, TN, C/N and soil pH appeared opposite trends, with mean TN decreasing from 1.92 to 1.40 g·kg^−1^, C/N declining from 12.08 to 9.61 and soil pH from 7.78 to 5.43 from north to south, respectively. Although most SOC fractions showed no significant differences among different sites (P > 0.05) , all labile carbon fractions exhibited a consistent north-to-south increasing pattern(Fig. 2), with POC ranging from 2.14 to 15.04 g·kg^−1^, DOC from 14.38 to 210.88 mg·kg^−1^, and MBC from 74.1 to 682.25 mg·kg^−1^, respectively. On the contrary, mineral-associated organic carbon (MAOC) showed an inverse distribution (4.86–24.81 g·kg^−1^), indicating the predominance of stable carbon fraction in northern soils, therefore implying a greater organic carbon lability in southern compared to the northern regions.

**Table 2:** Characteristic of sampled paddy soils.

Sample sites	TC (g kg^−1^)	TN (g kg^−1^)	C/N ratio	pH	Soil texture			NH₄^+^ (mg kg^−1^)	NO₃^−^ (mg kg^−1^)
					Sand (%)	Slit (%)	Clay (%)		
S1	11.33	0.88	12.88	5.66	41.11	40.07	18.82	6.71	0.21
S2	18.23	1.3	14.02	6.67	12.05	51.5	36.45	6.32	1.37
S3	30.2	2.24	13.48	5.49	17.63	49.28	33.09	8.18	1.76
S4	14.21	1.25	11.37	6.54	13.31	69.03	17.67	2.88	1.54
S5	12.24	1.1	11.13	6.33	38.85	49.88	11.27	6.57	2.56
S6	17.46	1.61	10.84	6.67	8.62	62.23	29.15	2.6	8.72
S7	15.06	1.39	10.83	7.12	15.74	64.74	19.53	1.55	2.42
S8	25.57	1.47	17.39	7.29	25.36	60.92	13.72	1.91	4.37
S9	16.97	1.74	9.75	7.11	15.15	70.76	14.09	0.91	7.29
S10	16.9	1.74	9.71	6.74	12.51	56.82	30.67	14.35	3.97
S11	15.39	1.89	8.14	6.75	15.46	52.55	31.99	21.88	1.72
S12	8.85	1.12	7.90	6.72	18.43	70.16	11.41	3.22	5.57
S13	24.49	2.32	10.56	5.89	16.16	56.34	27.5	27.16	5.25
S14	14.87	1.51	9.85	5.79	17.89	60.37	21.75	20.35	2.42
S15	10.3	1.04	9.90	7.78	7.99	67.99	24.01	9.2	1.19
S16	14.18	1.41	10.06	6.88	11.11	72.66	16.23	15.07	1.23
S17	26.34	2.65	9.94	6.52	17.44	63.17	19.38	5.16	28.11
S18	15.16	1.56	9.72	6.00	11.82	77.45	10.73	22.98	10.3
S19	31.86	3.24	9.83	6.17	7.43	64.81	27.76	24.49	17.3
S20	11.1	1.28	8.67	5.79	16.18	62.14	21.68	17.97	1.6
S21	18.13	1.96	9.25	5.81	17.74	54.28	27.98	18.28	2.18
S22	11.26	1.18	9.54	5.81	57.98	20.79	21.22	12.55	2.66
S23	15.53	1.52	10.22	5.91	23.75	55.89	20.36	23.39	2.42
S24	24.46	2.46	9.94	5.29	26.36	48.83	24.82	22.05	9.97
S25	12.77	1.35	9.46	5.59	32.66	33.67	33.67	22.54	2.88
S26	12.84	1.29	9.95	5.43	52.32	22.07	25.61	10.18	1.92
S27	20.19	2.24	9.01	5.74	17.75	41.8	40.45	14.94	24.64
S28	24.03	2.54	9.46	5.74	19.97	42.73	37.3	19.75	2.88
S29	23.29	2.24	10.40	5.43	61.04	20.89	18.07	16.65	2.42
S30	11.74	1.27	9.24	5.56	38.26	38.38	23.36	15.1	1.69

### Spatial patterns of CH₄ emission dynamics and cumulative emissions

**Fig. 3:**
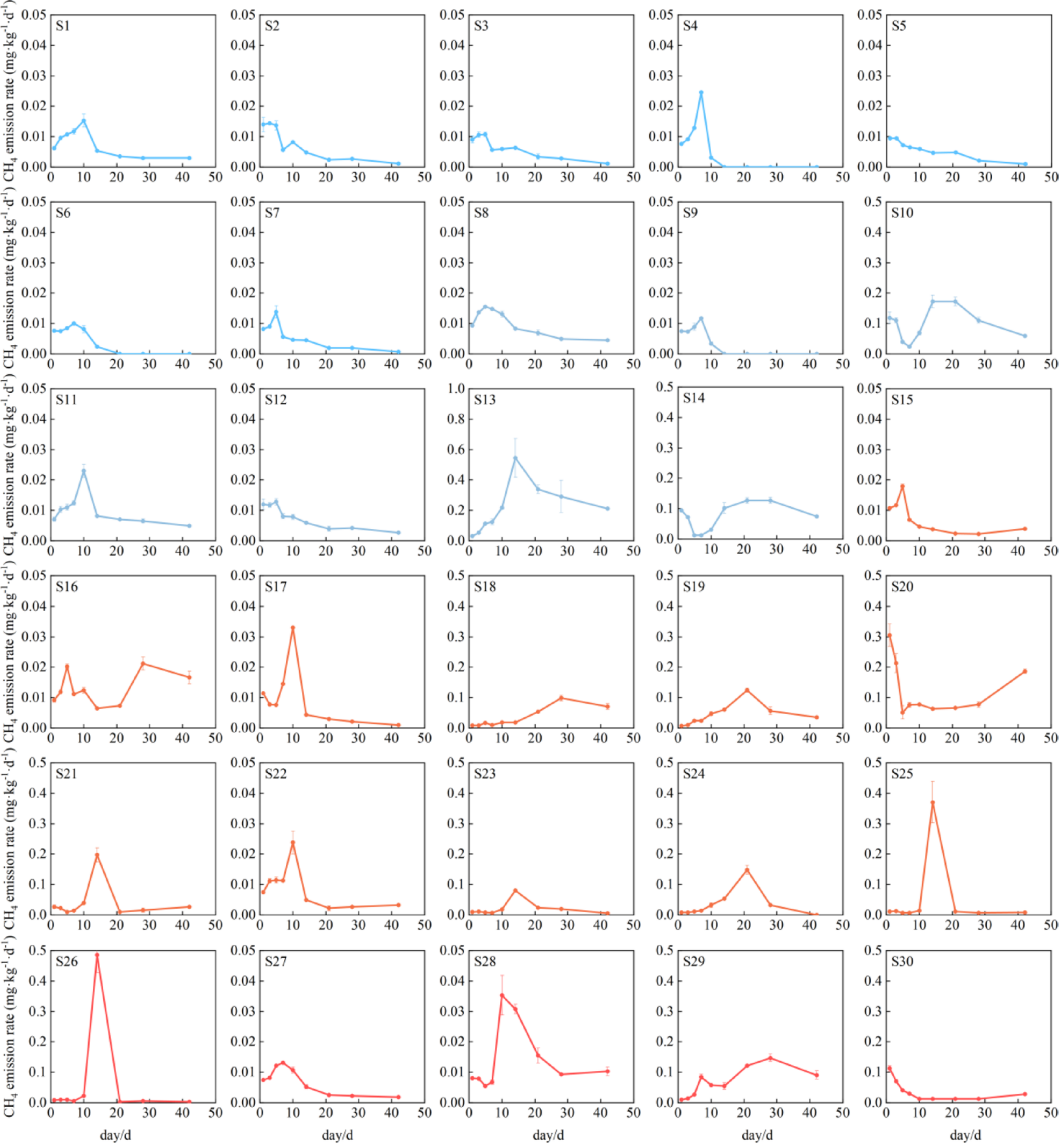
Variation of CH_4_ emission rates from different rice soils over time during cultivation.

**Fig. 4:**
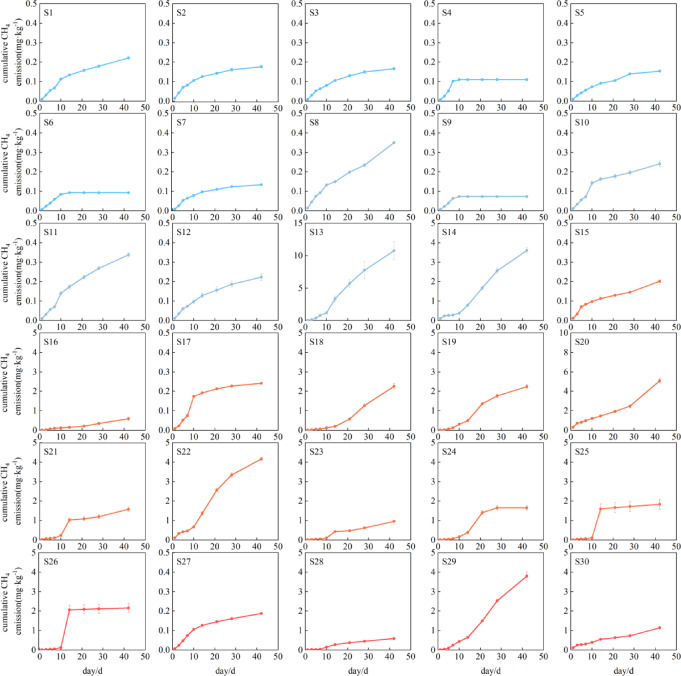
Temporal variation of cumulative CH_4_ emissions from different rice soils during cultivation.

**Fig. 5:**
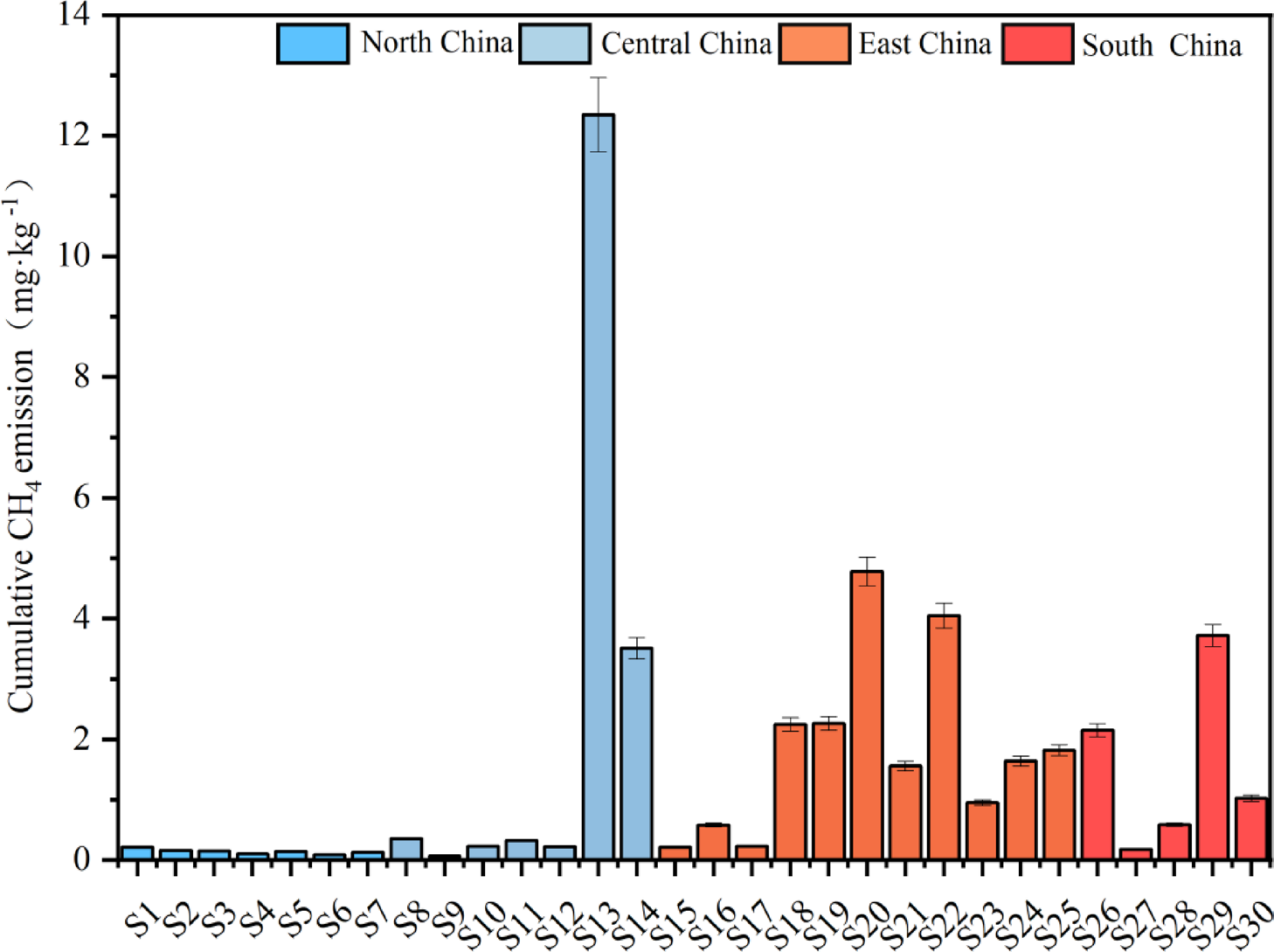
Cumulative CH_4_ emissions from different rice soils during cultivation.

To characterize the spatial pattern of CH_4_ emission, we measured the temporal dynamics of CH_4_ emission rates and cumulative CH_4_ emissions, and explored their spatial variability by comparing mean and peak value (Figs. 3–5). Peak CH_4_ emission rates in north China were significantly lower than those in central, east, and south China (P < 0.01), whereas no significant differences were found among central, east, and south China (P > 0.05). (Fig. 3). Specifically, mean emission rates ranged from 0.002 to 0.260 mg·kg^−1^·d^−1^, with several soils, such as S13 and S20, exhibited relatively high mean emission rates exceeding 0.10 mg·kg^−1^·d^−1^, whereas S9 showed the lowest mean emission rate. Most soils (83%) reached peak emissions during days 5–14. However, peak emission rates varied substantially over time (0.01–0.54 mg·kg^−1^·d^−1^), with 63% of soils exhibiting peak values below 0.1 mg·kg^−1^·d^−1^. S13 recorded the highest peak emission rate (0.54 mg·kg^−1^·d^−1^) and maintained elevated rates throughout the incubation period. In contrast, S26 (0.48 mg·kg^−1^·d^−1^) and S25 (0.37 mg·kg^−1^·d^−1^) showed transient peaks at day 14. In contrast, S5 soil exhibited the lowest peak (0.01 mg·kg^−1^·d^−1^) and a slow post-peak decline compared to other soils. These patterns highlight the possible complex interplay between regional climate–soil-microbe characteristics and methanogenic processes under flooded conditions.

**Fig. 6:**
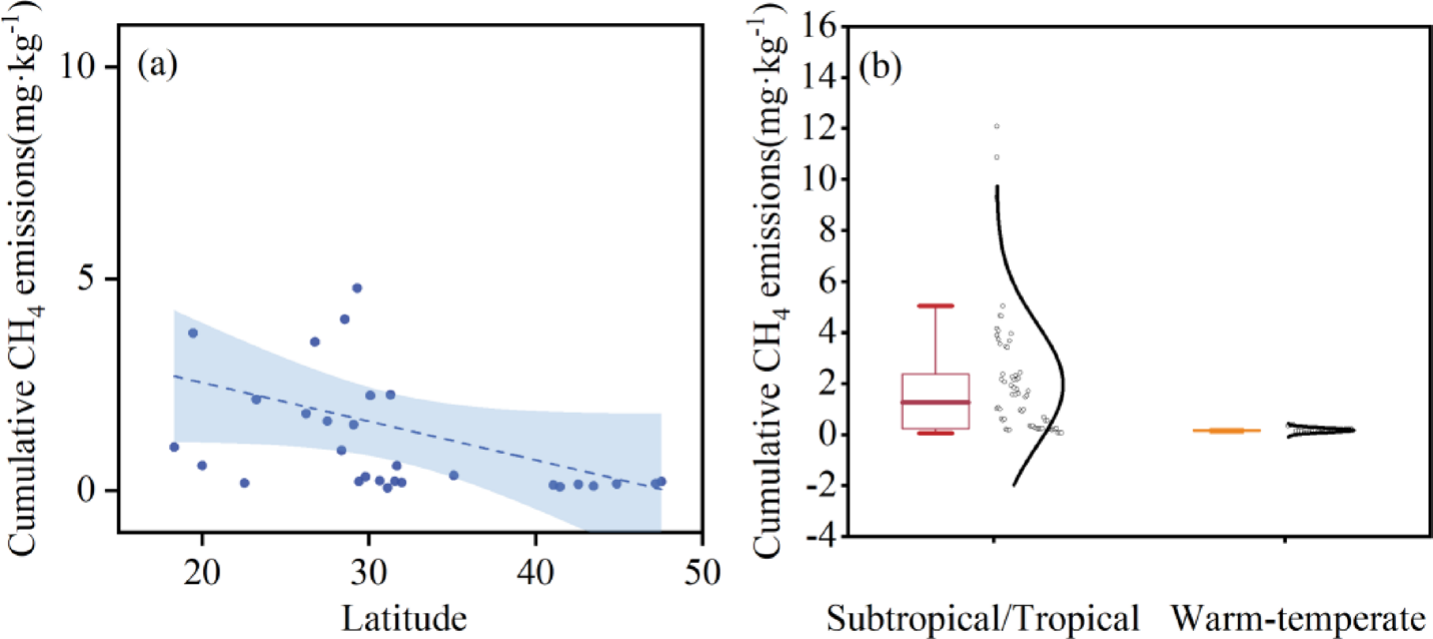
Variation of the cumulative CH_4_ emissions by latitude and climate zones.

Similarly, temporal variation of cumulative CH_4_ emission from the paddy soil samples showed spatial variability across different regions (Fig. 4), generally exhibiting a south-high, north-low geographical gradient, cumulative CH_4_ emissions in north China were significantly lower than those in central, east, and south China (P < 0.05), no significant differences were observed among central, east, and south China (P > 0.05) (Fig. 5). However, we did not observe significant latitudinal zonation patterns (0.08, P > 0.05)(Fig. 6). Specifically, among the sampled soils, 18 exhibited emissions below 1 mg·kg^−1^, predominantly clustered between 0 and 0.3 mg·kg^−1^, with S9 soil showing the lowest cumulative emissions. On the contrary, S13 soil shows exceptionally high emissions (10.75 mg·kg^−1^), surpassing S20 soil (4.78 mg·kg^−1^) by 2.3 fold and exceeding S9 soil by 168 fold. To further explore the spatial heterogeneity of cumulative CH₄ emissions, we categorized sampling sites into four sub-regions (north, central, east and south), ranging from 0.064 to 10.75 mg·kg^−1^. Among these regions, central China exhibited the most prominent cumulative CH₄ emissions (mean value: 2.44 mg·kg^−1^·d^−1^), followed by a secondary high emission in east China (mean value: 1.85 mg·kg^−1^·d^−1^). In contrast, south China showed relatively moderate and consistent cumulative emissions (mean value: 1.53 mg·kg^−1^·d^−1^), while north China had the lowest CH₄ emissions (mean value: 0.14 mg·kg^−1^·d^−1^) among all subregions.

Despite the distinct spatial heterogeneity observed in cumulative CH_4_ emissionis, no significant linear correlation with latitude was detected (Fig. 6a). However, we could observe a clear climatic zonation pattern, with tropical and subtropical soils consistently exhibited higher cumulative CH₄ emissions than temperate regions, showing statistically significant differences between climate zones (Fig. 6b). Cumulative emissions from tropical and subtropical soils ranged from 0.18 to 10.75 mg·kg^−1^ (mean value: 1.89 mg·kg^−1^), whereas temperate soils exhibited much lower emissions, ranging from 0.07 to 0.17 mg·kg^−1^ (mean value: 0.12 mg·kg^−1^). Temporal accumulation patterns also differed among sites. Most soils showed continuous increases over time. However, S4, S6, S9, S25, and S26 reached their maximum emission levels by days 10–14 of the incubation, followed by a stabilization phase. In contrast, S18 and S19 exhibited slower initial increases but accelerated emissions after day 14. Six soils (S13, S14, S18, S20, S22, and S29) consistently displayed the highest cumulative emissions and maintained faster accumulation rates toward the end of incubation.

### Environmental controls on CH₄ emission characteristics

**Fig. 7:**
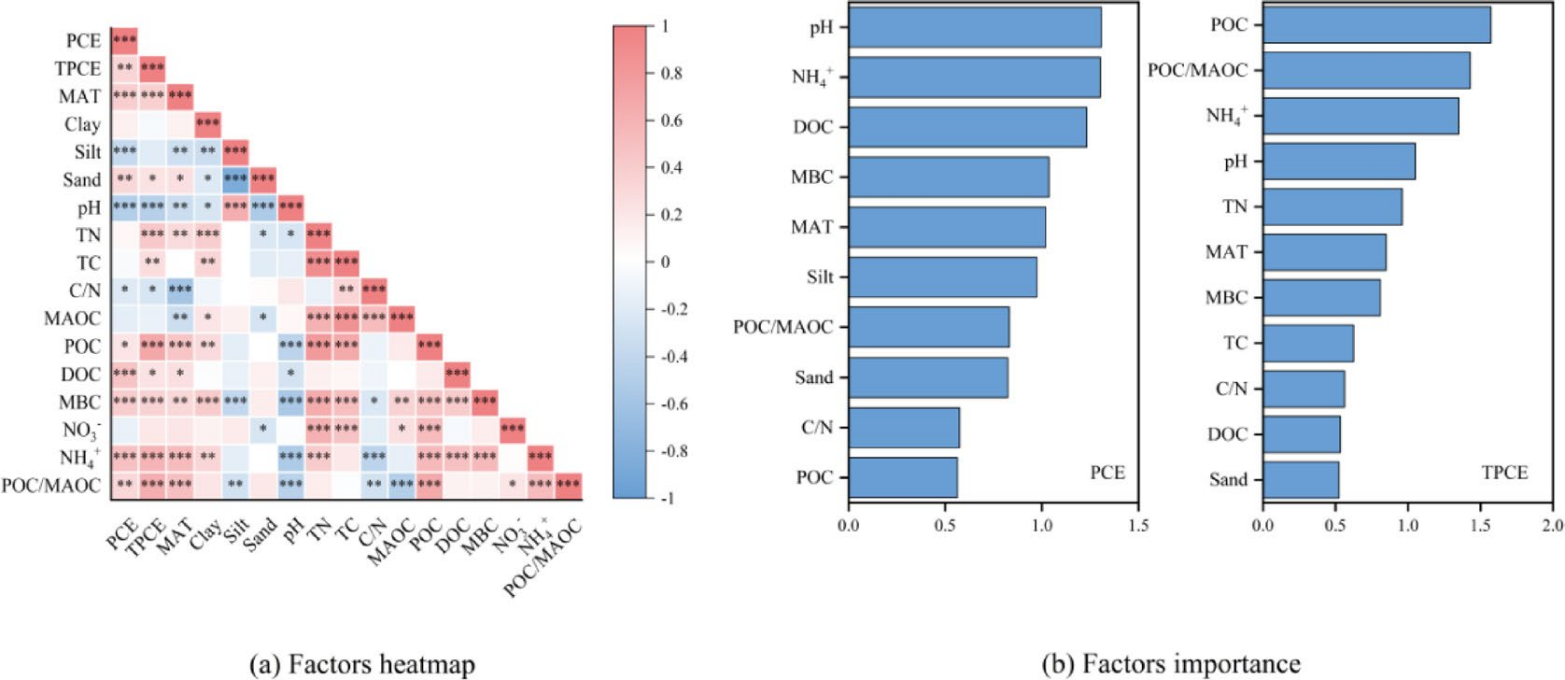
Factors and its importance analysis on the CH_4_ emission.

**Fig. 8:**
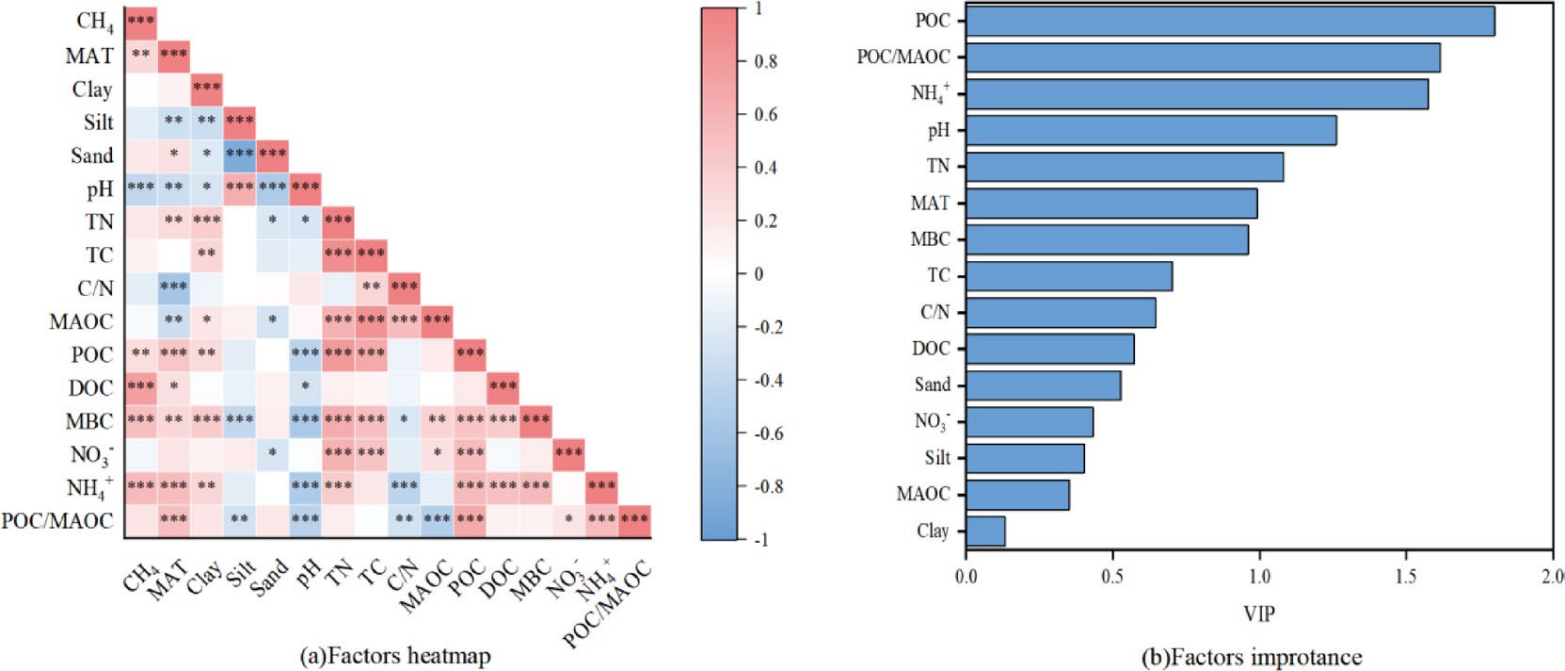
Factors and its importance analysis in the cumulative CH_4_ emissions.

**Fig. 9:**
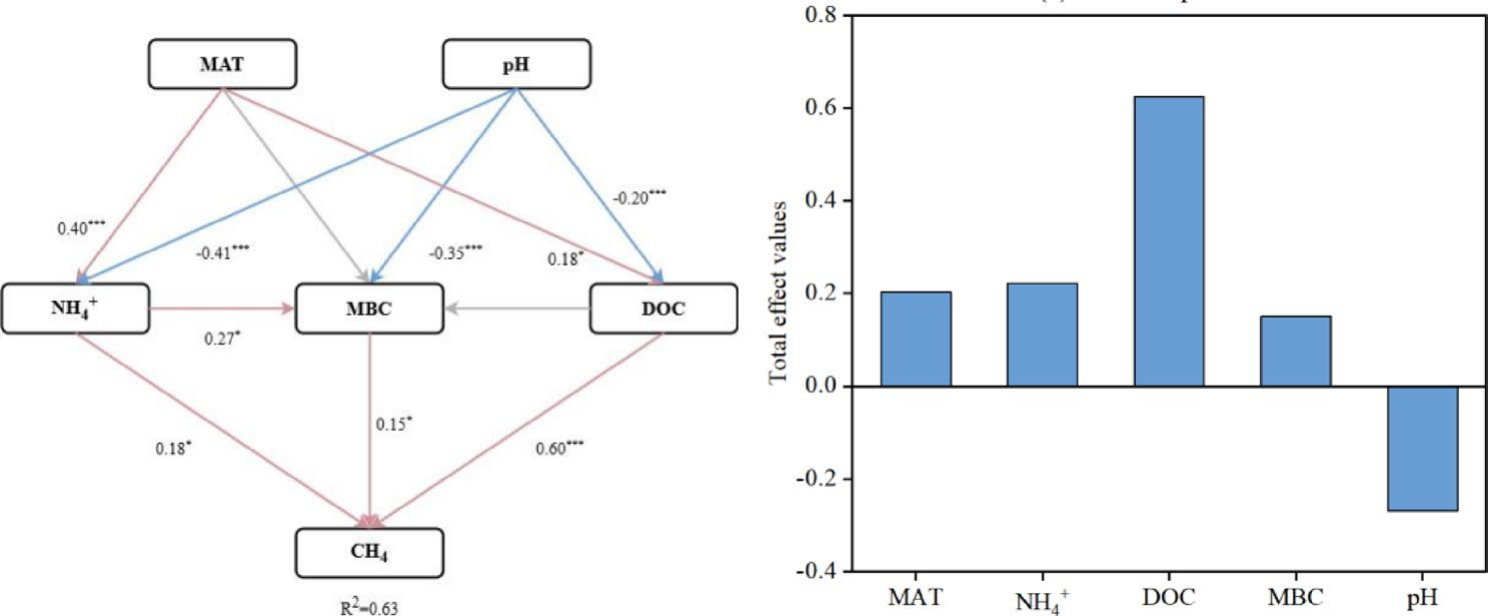
Factors and its importance analysis on the cumulative CH_4_ emissions.

To further investigate the underlying mechanisms, we conducted Pearson correlation analysis, PLS-DA model analysis, mixed linear models and SEM models, indicating that CH_4_ emissions were jointly regulated by climate, soil properties and carbon–nitrogen availability (Figs. 7, 8 and 9; Figs. S1-3). Firstly, we conducted Pearson correlation analysis and PLS-DA model analysis to investigate the important influencing variables. Pearson correlation analysis revealed that methane emission characteristics were significantly associated with MAT, soil pH, soil NH_4_^+^ content, soil texture, soil labile carbon fraction and POC/MAOC ratio (Figs. 7 and 8). Specifically, PCE were significantly positively associated with MAT, soil sand content, soil NH_4_^+^ content, POC, DOC, MBC and POC/MAOC ratio, and negatively associated with soil silt content, soil pH and soil C/N ratio (Fig. 7a). Time to peak CH_4_ emission rate (TPCE) was significantly positively associated with MAT, soil sand content, TN, TC, soil NH_4_^+^ content, POC, DOC, MBC and POC/MAOC ratio, and negatively associated with soil pH and soil C/N ratio (Fig. 7a). Cumulative CH_4_ emissions was significantly positively associated with MAT, soil NH_4_^+^ content, POC, DOC and MBC, and negatively associated with soil pH (Fig. 8a). PLS-DA model analysis further identified key drivers of CH_4_ emission characteristics in paddy soil samples (Figs. 7 and 8). Results revealed that peak CH_4_ emission rates were significantly regulated by soil pH, soil NH_4_^+^ content, DOC, MBC, MAT and soil silt content. Time to peak CH_4_ emission rates were significantly regulated by POC, POC/MAOC and soil NH_4_^+^ content (Fig. 7b). Cumulative CH_4_ emissions were significantly driven by POC, POC/MAOC, soil NH_4_^+^ content, and soil pH (Fig. 8b). To further clarify the direct and indirect regulators of environmental factors on the spatial pattern of CH_4_ emission, we conducted a SEM analysis (Fig. 9). The model explained 63% of the variance in CH_4_ emission (R^2^ = 0.63), revealing the direct influence of soil NH_4_^+^ content, MBC and DOC, and the indirect influence of MAT and soil pH, further implying the influence of climate–soil-microbe interactions in regulating CH_4_ emission spatial pattern. While direct pathways show DOC exerting the strongest positive effect on CH_4_ emissions (standardized path coefficient = 0.60, P < 0.001), and PLS analysis points to POC as the primary driver, the SEM conversely identifies DOC as the main driver. Considering our sample size and the well-established robustness of SEM for non-normal data, small-to-moderate samples, and complex environmental datasets in explaining overall variation and predicting outcomes^31,32^, we assert that DOC is the dominant driver. Soil NH_4_^+^ and MBC showed a weaker but significant positive direct effect (0.18 for soil NH_4_^+^ content and 0.15 for MBC, both P < 0.05). Soil pH did not directly affect CH₄ emissions but imposed a negative constraint on soil NH_4_^+^ content (-0.41, P < 0.001), MBC (–0.35, P < 0.01) and DOC (-0.20, P < 0.001). Similarly, rising MAT could significantly increased NH₄⁺ concentrations (0.40, P < 0.01) and DOC (0.18, P < 0.05), which in turn regulate CH₄ emissions, forming an indirect pathway enhancing CH₄ emissions. Collectively, these results indicate that soil N availability, microbial biomass and labile carbon availability together function as direct controls of CH₄ emissions, while climatic and edaphic factors regulate CH₄ fluxes indirectly by shaping microbial and substrate dynamics.

## Discussions

Across a broad latitudinal gradient in Chinese paddy soils, our results reveal a coherent spatial pattern of methane emission dynamics collectively driven by climate–soil-microbe variables. Labile carbon pools (POC, DOC, and MBC) consistently increased from north to south, whereas mineral-associated organic carbon (MAOC) exhibited an opposite pattern, reflecting contrasting mechanisms of carbon input, decomposition, and stabilization across climatic zones. These spatial patterns of carbon availability therefore cause spatial variance in CH₄ emissions, with peak emission rates, time to peak emission, and cumulative emissions differing by up to two orders of magnitude among sites. By jointly adopting Pearson analysis, PLS-DA analysis and SEM model analysis, we demonstrated that methane production in paddy soils is regulated not by a single dominant factor, but by the coordinated effects of carbon availability, nitrogen status, microbial activity, soil physicochemical constraints, and climatic context. More importantly, while PCE and TPCE capture short-term microbial responsiveness and substrate turnover kinetics, cumulative CH₄ emissions integrate these dynamic processes over time, providing a mechanistic bridge between intrinsic soil properties and large-scale spatial emission patterns. Together, our findings highlight that spatial heterogeneity in methane emissions emerges from the covariation of climate, soil carbon fractions, and microbial processes rather than from latitudinal temperature gradients alone, particularly under controlled anaerobic conditions, providing important implications for modulating carbon cycling processes under future scenarios.

### Spatial patterns of SOC fractions and CH_4_ emissions

We discovered that POC, DOC and MBC all exhibited a consistent north-to-south increasing pattern, while MAOC showed an inverse distribution (Fig. 2). The spatial pattern of labile carbon fraction is consistent with previous studies showing that warmer temperature and higher substrate availability would stimulate plant carbon inputs and microbial activities, thus accumulate fast-cycling SOC fractions such as POC, DOC and MBC^33–35^. On the other hand, unlike studies that reported weak or inconsistent climatic influence on MAOC^36,37^, our results reported a latitudinal decreasing gradient, with higher contents in northern China and lower in southern China. This may due to that soil in northern China under cooler temperature generally had lower decomposition rates, higher silt and clay contents, stronger mineral protection and higher organic carbon physicochemical stabilization, thus accumulated mineral-associated carbon^38,39^. Existing studies have shown that the spatial distribution of SOC in Chinese paddy fields exhibits significant regional differentiation characteristics, which are generally manifested in higher SOC contents in the paddy fields of south China, southwest China and the middle reaches of the Yangtze River than in those of north China and northwest China^40^. This distribution pattern is mainly co-regulated by climatic gradients, anthropogenic management measures and soil physical and chemical properties^41^. We further measured CH₄ emission rates and cumulative emissions and observed spatial variability, with mean emission rates spanning two orders of magnitude, peak emission rates varying by 54-fold and cumulative emissions by 168-fold, representing a south-high, north-low gradient. Such pattern are widely recognized in rice paddy soils, reflecting variance in soil carbon availability, redox dynamics, and methanogenic microbial potential^42,43^. At the regional scale, cumulative CH₄ emissions generally followed a south-high, north-low tendency, this is consistent with large-scale syntheses reporting higher methane emissions from warmer and more intensively cultivated regions of China^44^. The absence of a significant latitudinal pattern in our study likely arises from the controlled incubation design we adopted, which minimized the direct effect of spatial temperature and hydrological variance and thus emphasized intrinsic soil properties, mineral protection, and microbial functional composition variation.

### Regulators of peak CH_4_ emission rate and time to peak CH_4_ emission

Peak CH₄ emission rate (PCE) represents the maximum instantaneous intensity of methanogenesis under flooded conditions and reflects the short-term responsiveness of soil microbial processes to substrate availability and physicochemical constraints. In this study, Pearson correlation and PLS-DA analyses consistently identified soil pH, NH₄⁺ content, labile carbon fractions (particularly DOC), MAT, and soil texture as the dominant drivers of PCE, indicating that peak emissions are governed by the interaction between microbial metabolic potential and environmental controls (Supporting information Table S1). The strong positive associations of PCE with DOC, NH₄⁺, and MBC highlight the substrate-limited nature of methanogenesis in paddy soils, since DOC constitutes the most readily bioavailable carbon source for methanogenic archaea and can rapidly stimulate methane production by enhancing microbial metabolic rates^24,42,43,45^. Similarly, NH₄⁺ availability supports microbial growth and heterotrophic decomposition, indirectly promoting CH₄ production while suppressing methane oxidation through competitive and inhibitory effects on methanotrophs^46,47^. Together, these findings suggest that PCE primarily reflects the immediate coupling between substrate supply and microbial activity once anaerobic conditions are established. In addition to biological drivers, edaphic factors also imposed strong constraints on PCE. The negative relationship between soil pH and PCE observed here is consistent with previous evidence that alkaline conditions enhance NH₃ volatilization, thereby reducing NH₄⁺ availability and constraining microbial activity^48,49^. The effect of pH on cumulative methane emissions (PCE) mainly arises from its inhibition of the physiological metabolism of methanogens. Non-neutral pH disrupts the intracellular homeostasis of methanogens, alters their community structure and metabolic pathways, suppresses the enrichment of aceticlastic methanogens, reduces the transformation and utilization of methane precursors, and ultimately decreases PCE^50^. Soil texture further modulated peak emissions by regulating gas diffusion and water retention. Soils with higher silt content tend to restrict gas diffusion and delay methane release, whereas sandier soils facilitate CH₄ transport even under flooded conditions^48^. These results support the view that PCE integrates both biological potential and physical transport processes, explaining why peak methane fluxes are highly sensitive to multiple interacting soil properties rather than being controlled by a single factor.

TPCE captures the temporal dynamics of methanogenesis and reflects the time at which soils transition from initial anaerobic conditions to maximum methane production. Unlike PCE, which is dominated by instantaneous substrate availability, TPCE was primarily regulated by POC, the POC/MAOC ratio, and NH₄⁺ content, as revealed by both Pearson correlation and PLS-DA analyses (Supporting information Fig. S2). This indicates that TPCE is more strongly controlled by substrate persistence and decomposition kinetics than by immediately available carbon pools. POC represents a relatively slow-cycling carbon pool that can continuously supply fermentable substrates during prolonged anaerobic incubation, thereby sustaining methanogenesis over time^28,51^. Consequently, soils with higher POC content tend to exhibit delayed peak methane emissions, a pattern consistent with observations from long-term incubation and field studies showing that carbon quality strongly regulates methane production process^27^. The importance of the POC/MAOC ratio further emphasizes that the relative dominance of labile versus mineral-associated carbon pools influences the timing of peak emissions by regulating the rate at which substrates become available to methanogens. Nitrogen availability also played a key role in modulating TPCE. Elevated NH₄⁺ concentration stimulated microbial growth and accelerated organic matter decomposition, enhancing substrate supply for methanogenesis while extending the time required to reach maximum emission rates. This dual role of NH₄⁺ has been reported previously and highlights the tight coupling between carbon and nitrogen cycling in controlling methane dynamics^47,52^. Compared with PCE, TPCE therefore reflects the legacy effects of soil carbon composition and nutrient status, providing mechanistic insight into how substrate turnover rates shape methane emission kinetics rather than instantaneous emission intensity.

### Climate–soil-microbe together regulate cumulative CH_4_ emissions

While PCE and TPCE characterize distinct aspects of methane emission dynamics, cumulative CH₄ emissions integrate emission intensity and duration, providing a comprehensive measure of net methane production potential. Across Pearson correlation, PLS-DA, and SEM analyses, cumulative CH₄ emissions were consistently regulated by labile carbon fractions (DOC and POC), soil NH₄⁺ content, MBC, soil pH, and MAT (Supporting information Fig. S3).

Among these drivers, MBC reflects the size, activity, and metabolic status of soil microbial communities, which act as a core hub regulating methane emissions. Methanogen activity determines methane production, while methanotrophs reduce net emissions, their community structure, abundance and metabolic activity collectively control net methane emissions in anaerobic paddy soils^53^ (Chen et al. 2021). SEM identified DOC as the strongest direct regulator of cumulative CH₄ emissions, underscoring the central role of carbon availability in sustaining methanogenic activity throughout prolonged anaerobic incubation^19,20,54,55^. DOC not only serves as a direct substrate for methanogens but also supports microbial growth and metabolic activity, thereby maintaining methane production over time^24,42^. Nitrogen availability, represented by soil NH_4_⁺ content, increase the community abundance of methanogens and fermenting bacteria, but also competitively inhibit the key enzyme activities of methanotrophs and reduce the oxidative consumption of methane^47^ (Hu et al. 2015). In contrast, soil pH influenced cumulative emissions indirectly by regulating DOC, NH₄⁺ availability, and microbial biomass, consistent with previous findings that alkaline conditions constrain methanogenesis through substrate limitation^48,49^. A neutral to slightly acidic soil environment is more conducive to the enrichment and activity expression of methanogenic archaea, while reducing the adsorption and binding of DOC to mineral particles and improving substrate availability. In contrast, strong alkaline conditions inhibit the catalytic activity of microbial extracellular enzymes, disrupt the syntrophic relationship between methanogens and fermenting bacteria, and directly reduce methane production efficiency^56^. Climatic factors, particularly MAT, also exerted indirect control over cumulative CH₄ emissions by shaping substrate availability and microbial processes. Higher temperatures promote plant productivity and organic carbon inputs while accelerating nitrogen mineralization, thereby increasing DOC and NH₄⁺ availability in soils^52,57^. More importantly, a moderate temperature increase can significantly enhance the overall metabolic activity of the microbial community, accelerate anaerobic carbon and nitrogen turnover, and further strengthen the functional expression of methanogens. In contrast, high-temperature stress disrupts the structural balance of the microbial community, inhibits the expression of methanogenesis-related functional genes, and thereby reduces methane emission potential^53^. The spatial covariance of these climate, soil, and microbial variables collectively explains the observed south-to-north gradient in methane emissions across China, indicating that cumulative CH₄ emissions emerge from integrated climate, soil and microbe interactions rather than being driven by a single latitudinal factor^58^.

### Limitations and implications

Several limitations should be considered when interpreting our results. First, methane emissions were quantified under controlled incubation conditions, which effectively isolated intrinsic soil properties and microbial potentials but excluded in situ drivers such as plant-mediated transport, fluctuating redox conditions, and seasonal management practices that strongly influence field-scale methane fluxes^4,59^. Consequently, the absolute emission magnitudes reported here should be interpreted as potential rather than realized field emissions. Second, while our analyses identified DOC, NH₄⁺, and microbial biomass as key regulators of CH₄ dynamics, we did not explicitly characterize methanogenic and methanotrophic community composition or functional genes, which could further clarify biological mechanisms underlying regional variability. Future studies integrating microbial functional traits with SOC fractionation would improve mechanistic resolution. Finally, soil heterogeneity regulates methane emissions^60^. However, due to practical limitations, some initial soil properties were not determined in this study, which may introduce uncertainties in interpreting the mechanisms underlying the observed spatial variations in methane emissions.

Despite these limitations, our study has important implications for understanding and modeling methane emissions from paddy ecosystems. The strong control of labile carbon pools over cumulative CH₄ emissions underscores the importance of carbon quality, rather than total SOC alone, in regulating methane production potential. This finding supports emerging frameworks emphasizing SOC accessibility and microbial processing over bulk carbon stocks in predicting greenhouse gas emissions^34,36^. From a management perspective, practices that alter labile carbon inputs, nitrogen availability, or microbial activity—such as residue management, fertilization regimes, or water control—may substantially influence methane emissions without necessarily changing total soil carbon stocks. Incorporating these mechanistic insights into process-based models will improve predictions of methane responses to climate change and agricultural management across large spatial scales.

## Conclusion

This study provides a mechanistic understanding of the spatial variability of methane emissions from Chinese paddy soils across a broad climatic gradient. Across temperate to tropical regions, labile soil carbon fractions (DOC, POC, and MBC) increased whereas mineral-associated organic carbon showed an opposite pattern, indicating contrasting carbon stabilization and turnover mechanisms, contributing to differences in cumulative emissions which varied by over two orders of magnitude. Similarly, peak emission rates reflected short-term microbial responses to readily available substrates, while emission timing was more closely linked to carbon quality and decomposition kinetics. Structural equation modeling identified DOC as the strongest direct driver of cumulative methane emissions, with climate and soil pH exerting indirect effects through regulating substrate availability and microbial activity. Together, these results demonstrate that methane emissions from paddy soils are governed by coordinated climate–soil-microbe interactions rather than single dominant factors. Overall, our findings highlight the importance of carbon quality and microbial accessibility over total carbon stocks in driving methane emissions at large spatial scales, providing important implications for improving methane emission models and designing targeted mitigation strategies in rice-based agroecosystems under future climate change.

## Supplementary Information

Below is the link to the electronic supplementary material.

Supplementary Material 1

## Data Availability

The data supporting the findings of this study are available from the corresponding author upon reasonable request.
