# Supplementary material for: Methane emissions from rice paddies are regulated by carbon availability and soil pH along a mean annual temperature gradient
Source: Sci Rep. 2026 Mar 19;16:14129. doi: 10.1038/s41598-026-43940-8 (PMC13136294; doi:10.1038/s41598-026-43940-8)
Supplement: Supplementary file 1 — Supplementary Material 1 [file 41598_2026_43940_MOESM1_ESM.docx]

**Supporting information:**

**Characteristics of CH₄ emission and the mechanic from rice paddy soils across the climate zones in China**


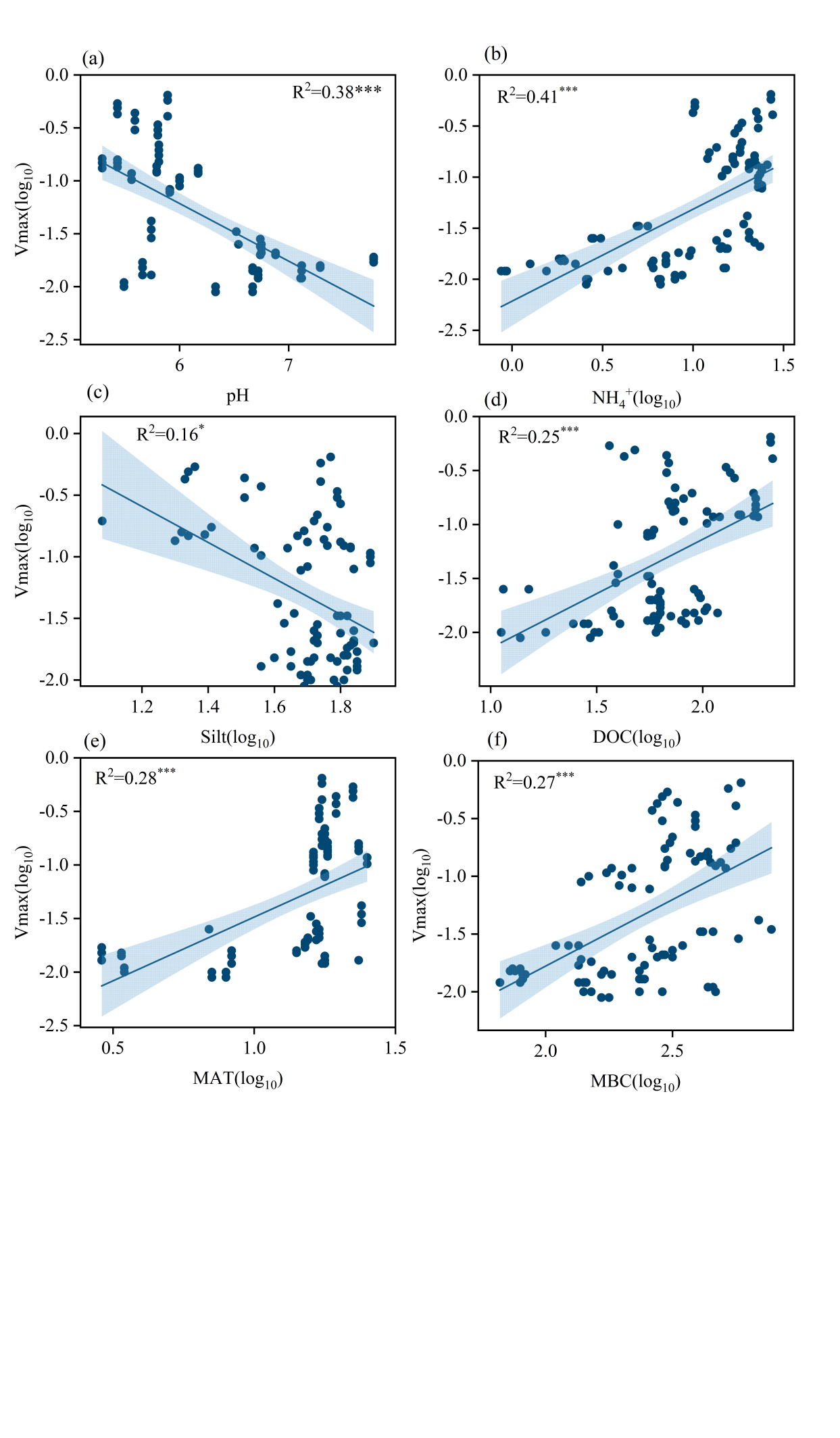


**Fig. S1 Relationship between the peak CH_4_ emission rate in paddy soil and various variables.(n=30)**


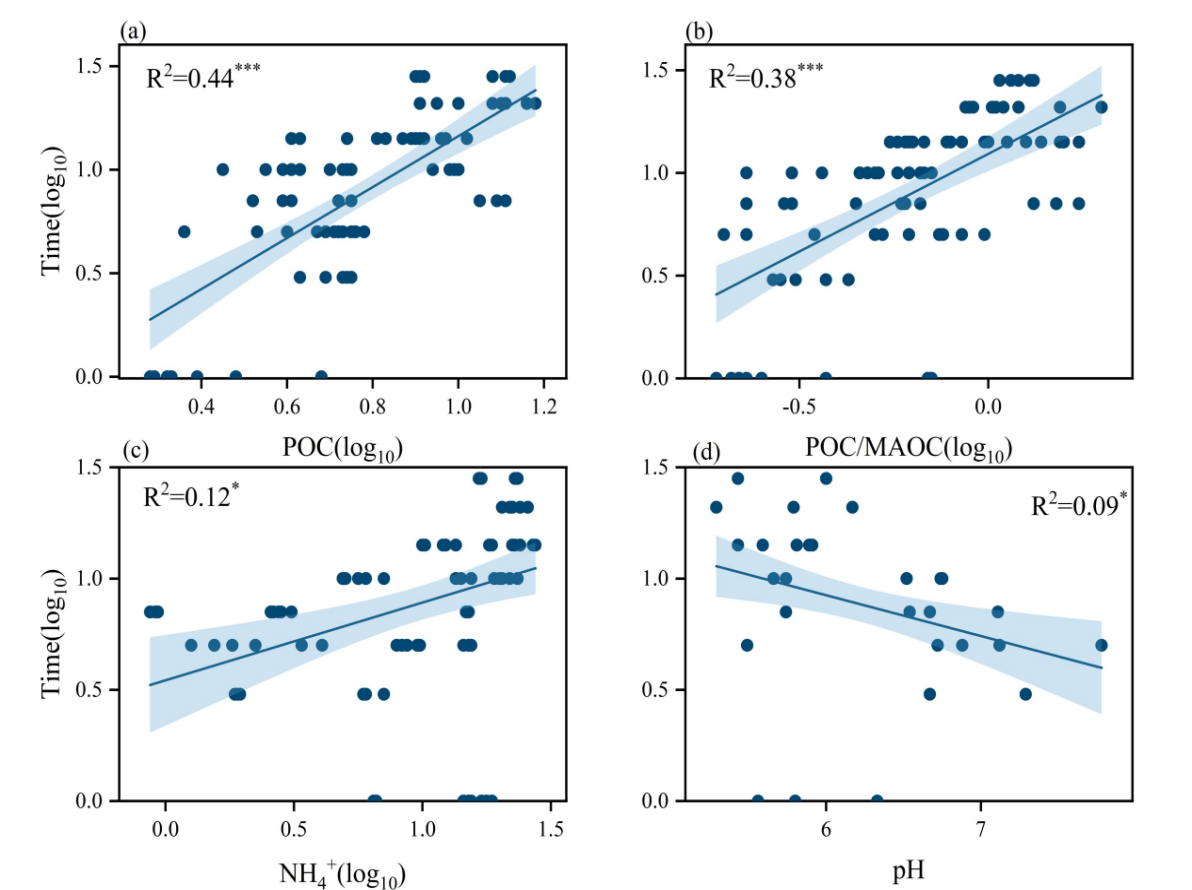


**Fig. S2 Relationship between time to reach peak CH_4_ emission rate and various variables in paddy soil.(n=30)**


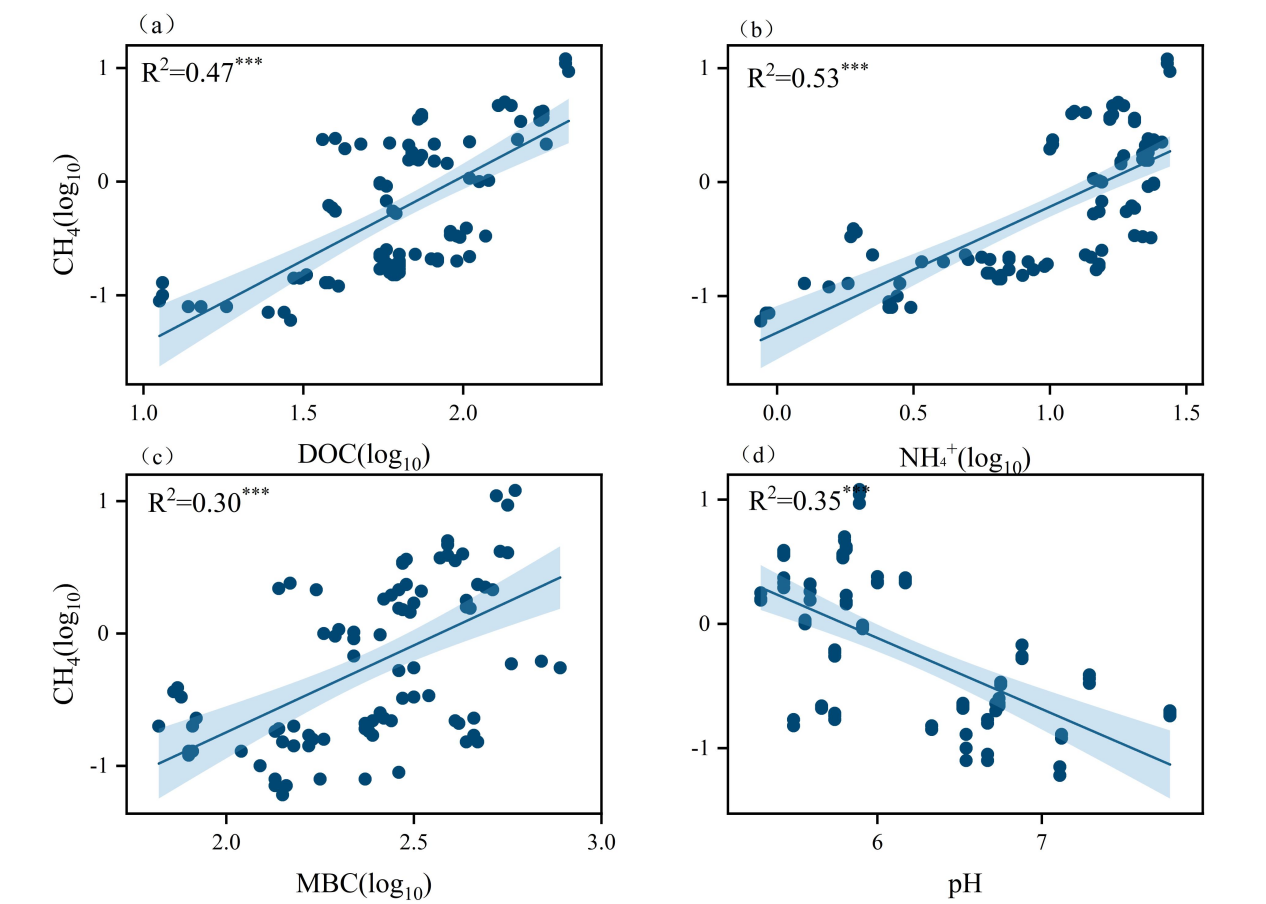


**Fig. S3 Relationship between cumulative CH_4_ emissions from paddy soil and its various variables.(n=30)**

**Table S1 Glossary of Abbreviations**

| Abbreviation | Full Name in English |
| --- | --- |
| α | Conversion factor from CH_4_ to C (12/16) |
| c | CH_4_ concentration |
| ρ | CH_4_ density at standard conditions |
| CH₄ | Methane |
| C/N | Carbon to Nitrogen Ratio |
| Clay | ClayMA |
| DOC | Dissolved Organic Carbon |
| *E* | Cumulative CH_4_ emission |
| FID | Flame ionization detector |
| m | Soil mass |
| MAOC | Mineral-Associated Organic Carbon |
| MAT | Mean Annual Temperature |
| MBC | Microbial Biomass Carbon |
| NH₄⁺ | Ammonium Nitrogen |
| NO₃⁻ | Nitrate Nitrogen |
| PCE | Peak CH_4_ emission rate |
| pH | Potential of Hydrogen |
| PLS | Partial least squares |
| POC | Particulate Organic Carbon |
| R | CH_4_ emission rate |
| Sand | Sand |
| SEM | Structural equation modeling |
| Silt | Silt |
| SOC | Soil organic carbon |
| t | Incubation duration |
| T | Incubation temperature |
| TC | Total Carbon |
| TN | Total Nitrogen |
| TOC | Total Organic Carbon |
| TPCE | Time to reach the peak CH_4_ emission rate |
| V | Headspace volume |
| VIP | Variable Importance in Projection |
